# Publishing your scholarship: a survey of pearls from top reviewers

**DOI:** 10.1080/10872981.2021.2016561

**Published:** 2022-01-07

**Authors:** Joshua Jauregui, Anthony R. Artino, Jonathan S. Ilgen, Gail Sullivan, Sandrijn M. van Schaik

**Affiliations:** a Department of Emergency Medicine, University of Washington School of Medicine in Seattle, Washington, USA; b Department of Health, Human Function, and Rehabilitation Sciences in Washington D.C., USA; c University of Connecticut School of Medicine in Farmington, Connecticut; d Department of Pediatrics in San Francisco, California, USA

**Keywords:** Scholarship, publishing, writing, journal reviews, survey, feedback

## Abstract

Experts have described ways to improve peer review quality. Perspectives *from* expert reviewers are largely absent in the health professions education literature. To gather guidance from expert reviewers, to aid authors striving to publish and reviewers aiming to perform their task effectively. This study surveyed *the Journal of Graduate Medical Education* (JGME) ‘Top Reviewers’ from 2017, 2018, and 2019. ‘Top Reviewers’ perform four or more reviews per year, with high average ratings. Top reviewers were sent an 11-item survey in February 2020. The survey included three demographic questions and eight open-ended, free-text questions about the concepts reviewers most often target in their reviews. We calculated descriptive statistics and performed a thematic analysis of open-ended responses. Of 62 eligible top reviewers, 44 (71%) responded to the survey. Only eight (18.2%) and seven (15.9%) respondents reported having ‘stock phrases’ or a reviewer template used for reviewer feedback to authors, respectively. The *what* (research question, methods), *how* (presentation, writing), and *why* (relevance, impact) were the resulting themes summarizing how reviewers categorized and responded to common problems. For ‘really good papers’ reviewers found the *what* acceptable and focused on *how* and *why*. For ‘really bad’ papers, reviewers focused on big picture feedback, such as the value of the study. Top reviewers from a single health professions education journal appear to have similar approaches to conducting reviews. While most do not use stock phrases or templates, they share similar strategies to differentiate ‘good’ vs. ‘bad’ papers through the *what, why*, and *how* of a manuscript.

## Introduction

Peer review is integral to the dissemination of high-quality scholarship in health professions education (HPE). As informed members of a journal’s audience, peer reviewers share their methodological and content expertise to provide meaningful feedback to authors. Such feedback can help authors substantially, to clarify an article’s narrative, enhance the rigor of its methods, and broaden the scope of its conclusions [1]. Furthermore, peer reviews are a key factor in editorial decisions [1–4]. Although authors may disagree with some elements of reviewers’ feedback, incorporating feedback ultimately strengthens the authors’ work [1–5]. Given the increasingly competitive nature of HPE publishing, a richer understanding of what reviewers are looking for could provide anticipatory guidance to authors. In addition, considering the increasing number of submissions to medical education journals, there is a dire need for reviewers who can provide high-quality reviews [6]. Thus, insights into how experienced reviewers approach the review process can help new reviewers develop effective strategies.

Much has been written about ways to improve the quality of peer review, generally through highlighting the characteristics of high-quality reviews from the perspective of journal editors and experts in the field [1,7–9]. These articles are typically framed as lessons *for* reviewers, with an aim to optimize the peer review process. Perspectives and resulting guidance *from* expert reviewers are largely absent in the HPE literature. The few reports that examine peer review from the reviewer’s perspective do so indirectly by analyzing reviewers’ comments and manuscript characteristics associated with rejection [10–12].

Our objective was to obtain the perspectives of reviewers who consistently produce high-quality education journal reviews. We aim to gather useful guidance from expert reviewers, to aid authors striving to publish HPE scholarships and reviewers who are new to the peer review process.

## Methods

### Design and population

This study used a cross-sectional design to survey all ‘Top Reviewers’ from the Journal of Graduate Medical Education (JGME) during the 2017, 2018, and 2019 calendar years. We chose to study a purposeful sample of JGME reviewers given their deliberate approach to selecting top reviewers based on the quality of their reviews and our access to the study population [13]. The top reviewers are chosen by the JGME editorial team each year from a pool of reviewers who have performed four or more reviews during the year based on internal quality metrics of all JGME reviews. Review quality ratings are assigned for each written review by the editor-in-chief, with input from the deputy and associate editors. The rating range is 51 to 100. During the years included in this study, ratings of reviews by top reviewers averaged 85 (range 70 to 98) with 29% of reviews scoring 90 or more points. We contacted potential participants on 3 February 2020, with emails that included a link to the survey instrument and recruitment information, which emphasized that participation was anonymous, voluntary, and without compensation. Two reminder emails at one-week intervals were sent thereafter. We completed the data collection on 18 February 2020.

### Survey content, validity evidence, and protocol

We iteratively developed an original, 11-item survey instrument using Messick’s unified theory of validity [14]. We collected initial validity evidence using two sources: evidence based on content and evidence based on cognitive processes. We determined the initial survey content based on the expertise of the authors (JGME reviewers, a deputy editor, an associate editor, and editor-in-chief), guided by the study aims (Appendix) [15]. The survey instrument included eight open-ended, free-text questions about reviewers’ approaches to peer review and the aspect reviewers most frequently addressed in their reviews. We asked reviewers to describe their approach for manuscripts they deemed to be ‘really good’ versus those they deemed to be ‘really bad,’ to solicit salient aspects on either extreme. We asked authors to specifically describe ‘stock phrases’ or recurring language they use in their reviews, if applicable. We also included three questions about participant demographics.

Next, we collected validity evidence based on cognitive processes. To this end, we performed ‘think aloud’ interviews, which allowed us collect evidence of response process validity and to improve item clarity [16]. We conducted these interviews with two JGME reviewers who are colleagues of the first author (JJ) and who did not meet criteria for inclusion in the study as top reviewers. We then piloted the survey with two additional, different individuals – a PhD education scientist and deputy editor of *Academic Medicine*, and a PhD in measurement, assessment, and evaluation with a postdoctoral fellowship in medical education and qualitative research methods. We incorporated feedback from both individuals into the final version of the survey instrument, which we determined through consensus of all authors (see Appendix).

### Data collection and analysis

None of the authors were participants in the survey. We collected and managed the study data using REDCap, an electronic data capture tool hosted at the University of Washington. REDCap (Research Electronic Data Capture, Nashville, Tennessee) is a secure, web-based software platform designed to support data capture for research studies.

We conducted our data analysis using Microsoft Excel 2018 (Redmond, Washington), to calculate descriptive statistics: percentage of respondents, mean *(M*) and standard deviation (SD) of items as appropriate. We numbered all participants by the order in which they completed the survey. We conducted a thematic analysis of the open-ended responses using Microsoft Word 2019 (Redmond, Washington) and analytical memos [17]. As JGME reviewers and editors, we paid particular attention to how these identities affected how we interpreted the data. Professionally, our author group was intentionally composed of three MDs, a PhD, and an MD/PhD to reflect the professional heterogeneity of reviewers. We used a constructivist approach to our inductive data analysis. This approach acknowledges that meaning is created through the interaction between the investigators and participants. All authors read all responses and then met to discuss salient findings. We then divided into two groups [(JJ and SV) and (AA, JI, and GS)] to independently code the responses, line by line, and met in our groups to compare and reconcile coding, and to identify and categorize the main ideas. JJ and SV then met to review the categories identified by both groups and through discussion refined the categories and grouped them into major themes.

The Human Subjects Division at the University of Washington deemed the study to be exempt from review.

## Results

Of 62 eligible JGME top reviewers, 44 (71%) responded to the survey. Respondents reported that they had been a reviewer for HPE journals for, on average, 10.4 years (SD = 8.5 years). Thirty-nine (88.6%) respondents reported that they worked at an academic institution at the time of the survey distribution and the remaining five (11.4%) had done so previously. Because reviewers’ lived experiences may influence their responses, we provide additional demographic data in Table 1. Specific quotes are noted by participant identification numbers.

**Table 1.** Demographics of respondents

Survey Item					
How many years have you reviewed for health professions education (HPE) journals?	Mean	Standard Deviation			
	10.42	8.48			
What is your affiliation with an academic institution?	Currently work in an academic position	Formerly worked, but no longer works, in an academic position			
	39 (88.6%)	5 (11.4%)			
What is your gender?	Female	Male	Non-Binary	Did not answer	
	24 (54.5%)	15 (34.1%)	1 (2.3%)	4 (9.1%)	
Of the following, what degrees do you hold (select all that apply)?	Bachelor’s degree	Master’s degree	Medical Doctoral degree	Non-Medical Doctoral degree	Other professional degrees
	29 (65.9%)	21 (47.7%)	36 (81.8%)	6 (13.6%)	2 (4.5%)

Only eight (18.2%) respondents reported having specific ‘stock phrases’ and/or paragraphs they used repeatedly in their reviewer feedback to authors, while 36 (81.8%) did not. Similarly, only seven (15.9%) respondents said they used a reviewer template when working through their peer review process, while 37 (84.1%) did not.

We identified three themes that describe the common problems JGME reviewers encountered when reviewing manuscripts and the ways in which they responded to such problems in their reviews. We describe these three themes as the *what*, the *how,* and the *why* of the problems identified (Table 2). The *what* encompasses the question addressed and the methodology of the study. The *how* refers to the ways in which the question and methodology are presented in the paper, and the *why* is the relevance and impact of the study. Reviewers indicated that their specific response to the *what, how*, and *why* depended on their overall perceptions of the quality of a paper: they differentially focused on each of these elements depending on their initial holistic review.

**Table 2.** Major themes

Major Theme	Definition	Category of Concern	Representative Quotes
What	The research question addressed, the choice of methods and implementation of those methods	Insufficient literature review	‘The research design is flawed. At times the authors intend to contribute to an important teaching or educational issue but have not approached the problem in such a way that the collected data is valid or reliable, so the whole project may lack rigor or quality and therefore lack meaningful conclusions.’ (30) ‘The use of tools that have not been previously examined leads to repeating work or coming up with answers that cannot be applied to other settings or integrated with other research.’ (13) ‘Poor evaluation of educational programs; the paper describes an educational program for residents (or occasionally faculty) but does not adequately evaluate the effectiveness of the program in changing practice or attitudes.’ (28)
		Methods did not match study question	
		Mistaking association for causation	
		‘Low level’ or ‘mismatched’ outcomes (e.g., learner satisfaction)	
		Insufficient limitations (needs to be deliberately linked to methodological approach)	
		Poor measurement or lack of validity evidence	
How	The organization, presentation, and articulation of the study	Writing/organization of poor quality	‘Inadequate description or definition of key ideas, principles, or findings that will allow for understanding by a larger audience.’ (7) ‘Overstated conclusions, overstated aims. People either draw very arching conclusions that are not supported by their data or set out to answer massive questions and then have conclusions on only a small part of that.’ (12) ‘Obfuscation with words that may be pretty but contribute nothing. I think that ideas of importance sufficient to merit publication should not challenge the reader’s ability to digest content.’ (26)
		Unclear study goals	
		Reporting of methods lack structure or methodology isn’t clear/transparent	
		Poor data analytics	
		Misinterpretation or misuse of statistics	
		Too much or too little in results	
		Does not offer heuristic in discussion to connect to the field (implications for educators, implications for researchers)	
		Over-interpretation of results/Conclusions not supported by results	
Why	The relevance and impact of the study	No clear argument for why the study is important (‘problem, gap, hook’)	‘Build a logical story. What is known on this topic, what do we need to know (what are the research gaps), what we did to fill these research gaps.’ (10) ‘The main problem is not having a theoretical framework for the research. This could be point 2 and 3 as well. It is so important and almost always missing.’ (39) ‘Unclear justification for why the study was needed (or lack of logical argument).’ (41)
		Lacking or unclear conceptual/theoretical framework	
		Results not relevant or new, not applicable to others/GME, too narrow in scope	
		Importance of findings unclear, not linked to literature	

When reviewers perceived a paper as ‘really good,’ then, typically, they had already bought into the *what* and focused on optimizing the *how* and *why*. For example, one participant reflected: ‘When the bones of a paper are great, I can focus on the story that is being told and things like readability and flow. This is much more difficult when there are lots of flaws in the actual approach.’ (15) Occasionally, reviewers commented on specific details of the methods, in ways that they would not typically do if a paper was ‘really bad.’ For the *how*, reviewers often gave feedback on the writing style, the organization of the paper, and the overall story arc. For the *why* they tended to give the most feedback on how authors could better articulate a study’s generalizability or transferability and better explain its impact on the field.

When expert reviewers perceived that a paper was ‘really bad,’ they tended to focus on big picture feedback, such as the value and impact of the study, rather than the details. They often commented on the paper’s overall strengths and weaknesses first, and then followed with feedback on some of the minor details. They often took an empathetic and generous stance such as ‘I assume that my best friend is about to get this review’ (9) and placed positive feedback first. They also prioritized certain parts of the review when they had many concerns about a paper, by highlighting areas that were problematic. While this feedback often focused on the *what* (research question and methodology) or *why* (relevance and impact), reviewers recognized that the *what* and *why* are difficult to assess if there are major issues with *how* a study is presented. In other words, if a paper is disorganized or otherwise not clearly written, it limited reviewers’ interpretations of what was done and the study implications. In general, when reviewers found what they perceived to be fatal flaws, reviewers focused on being very direct and kept comments short. However, comments from our respondents indicated that most of the JGME manuscripts reviewed had fixable issues rather than fundamental flaws.

## Discussion

Our small study reveals that many of JGME’s top reviewers have similar approaches to conducting reviews although few use stock phrases or a template. Focus areas of feedback can be grouped under *what* (research question and methodology), *how* (organization and presentation) and *why* (relevance and impact), and the extent to which reviewers address each of these areas depends upon the overall perceived quality of a paper. Reviewers often limit the number of comments for papers considered ‘really bad,’ which may reflect an emphasis on efficiency, yet also describe taking an empathetic stance.

There are many useful resources to guide authors in preparing a manuscript for publication. Aside from instructions for authors provided by individual journals, helpful resources include tips from editors, the ‘writers’ craft’ series by Dr. Lorelei Lingard, and Dr. Geoff Norman’s ‘12 tips’ paper on how to ensure your paper does *not* get published [18–20]. There are even papers providing guidance to authors whose paper is rejected [21]. The present study adds to these resources by offering authors insights into one journal’s top reviewers’ thought processes and paper review approaches. Our study highlights the imperative of clarity and organization, as major issues in this area may make it impossible for reviewers to formulate determinations about the quality or relevance of the work described. Thus, clarity and organization are almost a sine-qua-non for reviewers to provide useful feedback about the actual work described in a paper. Our findings also emphasize that a paper should not only clearly describe what was done but also discuss the relevance and impact of the work in a compelling way. These recommendations align well with the advice given by Lingard and Watling (2021) in their recently published book entitled *Story Not Study: 30 Brief Lessons to Inspire Health Researchers as Writers*. Their central thesis is that ‘Great research papers, like great stories, are compelling, memorable, and persuasive. They grab and hold readers’ attention, increasing the odds that the research findings will reach and influence their intended audience’ (p. 1) [22].

The top reviewers in our study reported that most submitted manuscripts had fixable issues and would be publishable. We therefore encourage authors to review their work carefully prior to submission, considering first and foremost the clarity of writing. Having a colleague outside the research team read the manuscript, with the how, what, and why framework in mind, can be useful to anticipate reviewer feedback and optimize chances for acceptance.

Many journals have clear guidelines for reviewers, which indicate the areas for preferred attention [23,24]. Yet, few reviewers receive formal guidance or training on how to conduct a review. While some journals offer written feedback for reviewers, this practice does not appear to be the norm [5,25]. Other useful published resources for reviewers include a tips paper that offers new reviewers guidance on the overall process and an editorial with suggestions on constructive language and tone [8,26]. Because lack of formal training in how to conduct a high-quality review appears to be an existing gap, graduate degree programs in HPE, which are growing in popularity, might consider including reviewer training in their curricula.

The results of our study provide insights for new reviewers as well as concrete suggestions for a structured approach to the organization and prioritization of reviewer comments. After reading a manuscript, a reviewer can focus first on whether the overall research question and methods (*what*) are of sufficient quality. If not, the reviewer can decide whether to invest additional time in providing detailed feedback regarding the writing and organization (*how*) – the story – and the overall relevance and importance of the work (*why*). This strategy is likely to help the reviewer work more efficiently. The different categories in each of the themes (Table 2) can serve as a checklist for reviewers, especially if a journal’s reviewer instructions are non-specific.

The study findings are limited by our use of a short survey and a single journal. This makes generalizing or transferring our findings to other journals or settings difficult. In addition, we limited our survey to top reviewers, defined by one journal, who comprise a small fraction of all reviewers. This skews the results towards this small group, and while they may be consequential by the nature of their standing as top reviewers, they are not necessarily representative of expert reviewers for other HPE journals. Furthermore, while we intentionally refined our survey with specific steps to establish validity evidence and minimize undue bias, the initial survey was developed by authors’ consensus, which potentially limits the scope of the questions. We did, however, include several open-ended items, which allow respondents to offer new content. Because of our purposeful sampling strategy, this led to a rich data set with in-depth information about respondents’ approaches to the review process.. Finally, despite attention to rigor in our approach, the findings are limited by the quality of the qualitative data that can be gathered from open-ended survey questions, in which follow-up questions, to improve clarity and depth, are not possible [27].

Future study of processes used by reviewers who consistently produce high-quality reviews, as defined by authors as well as editors, may provide additional insights for those who wish to improve the quality and efficiency of their reviews. Further work could also explore the impact of high-quality reviews on authors. While one hopes that high-quality reviews will help authors improve their work for resubmission as well as future manuscripts, to our knowledge there is no empirical evidence to support whether this is true and, if so, which review elements are most useful to authors.

## Conclusion

Top reviewers for a single, health professions education journal have similar approaches to conducting reviews. While they do not tend to use stock phrases or reviewer templates, they do share a similar focus on the *what, why*, and *how* of a manuscript. For ‘really good’ papers, top reviewers focus on improving the paper in areas of *how* and *why*: writing, organization, relevance, and impact. In contrast, for ‘bad’ papers, which have problems with the research question or methods, reviewers typically provide brief comments and may use an empathetic tone.
